# Activity-Based Approach to the Teaching and Psychology of Insightful Problem Solving: Scientific Concepts as a Form of Constructive Criticism

**DOI:** 10.11621/pir.2023.0302

**Published:** 2023-09-15

**Authors:** Alexander N. Romashchuk

**Affiliations:** a Lomonosov Moscow State University, Moscow, Russia

**Keywords:** activity theory of learning, conceptual change, naive concepts, scientific concepts, constructive criticism, insightful solution, full insight

## Abstract

**Background:**

This article is dedicated to the 100-year anniversary of the birth of N.F. Talyzina and contains an assessment of the prospects for developing ways to master a scientific concept, in theories of learning according to the activity approach. The assessment takes into account achievements following the approach of L.S. Vygotsky, the “conceptual changes” approach, and theories of the psychology of insightful problem solving.

**Objective:**

To demonstrate the necessity and productivity of the activity approach to scientific concepts that students learn as forms of constructive criticism.

**Method:**

A comparative analysis of the theories of the activity approach to learning, the approach of L.S. Vygotsky, the “conceptual changes” approach, and theories of the psychology of insightful problem solving, from the standpoint of identifying the most effective way of mastering scientific concepts.

**Results:**

The main substantiated thesis of the article is that mastery of a scientific concept is most effective when it is presented as a form of constructive criticism of another concept.

**Conclusion:**

Taking into account the conceptual forms of constructive criticism allows us to outline the actual paths of development of the activity approach to learning. These forms were developed, on the one hand, through the methodology of science, and on the other, in a less developed way, through the psychology of insightful problem solving, with reliance on certain forms of “critical” action. In particular, when using a special kind of obstacle to teach a task, it is proposed to use the analysis of “full insight” to reveal a special type of reason for an erroneous action.

## Acknowledgment

While I was still a graduate student, friends from the laboratory of educational psychology recommended me to the head of the laboratory for the vacant position of engineer, and N.F. Talyzina, after giving me a test assignment, took me on for this position. During my work in the laboratory, Nina Fedorovna taught me a number of most important lessons for professional life, one of which is directly related to the topic of this article.

With all the almost mathematical clarity and rigor of N.F. Talyzina’s theory, when discussing the reports of the laboratory staff, she demonstrated amazing flexibility, lively interest in discussing different approaches, and welcomed alternative theories and views. She showed great wisdom in everything, including when we visited her hospitable home, and performed some small work assignments, which imbued all the activity in the laboratory with a sense of trust. This characteristic of Nina Fedorovna, her creation of an atmosphere of free discussion, is close to the main topic of this article: constructive criticism. Out of gratitude to her for those creatively active and free years in the laboratory, in her memory, I would like to designate this article as another report presented at a laboratory meeting, a farewell report.

## Introduction

For various theories of the activity approach to learning, one of the unresolved problems is the ambiguous answer to the question of what relationship newly formed concepts have to concepts already existing in the student’s mind. Let us first discuss the content and meaning of this problem.

Galperin most succinctly outlined the specifics of his theory during his lectures on psychology, first in separating the structure of an object and the structure of action with the given object, and, second, in his emphasis in teaching on the means by which the action is carried out and which correspond to the structure of the object (Galperin, 2002). Talyzina endorses these positions in her approach: “The fundamental difference between our approach to the study of the process of concept formation and the previously considered one [that of L.S. Vygotsky] lies in the fact that, first, we study this process from the aspect of activity, of actions associated with the formation and functioning of concepts. Second, the formation of actions associated with a concept can be traced not in conditions of spontaneous mastery, but in conditions of comprehensive control over the course of their formation” (Talyzina, 2018, p. 191). According to Talyzina, all this control is aimed at combating formalism, in particular, the “verbalism” of knowledge, which she partially reproaches even Vygotsky for not having sufficiently overcome (Talyzina, 2018, p. 188).

Control of the formation of an action presupposes, first of all, a fully generalized orienting basis of action. In turn, the completeness of the orienting basis of action leads to the idea that concepts are adequate not individually, but only in a system with other concepts. The generalization of action is most often understood as the possession of more general methods of cognitive activity, which serve for the derivation of individual concepts. For example, the action of summarizing concepts is considered by Talyzina as the basis for deriving not only mathematical concepts, but all scientific concepts that have a conjunctive structure of attributes in their definition (Talyzina, 1995). And this derivation is considered the main method of overcoming formalism in the mastery of a concept (Talyzina, 1995).

Despite all their differences in the understanding of learning with V.V. Davydov, another representative of the activity approach, such theses as the derivability of a learning concept, its activity basis, and the systemic nature of the concept unite his theory with those of Talyzina and Galperin (Davydov, 1996). All three theories have one more common feature, which is that the system of concepts is formed, as it were, from a “clean slate,” and is not, for example, transformed from another system of the student’s concepts. This feature and its significance can be considered most conveniently on the basis of the relationship between naïve and scientific concepts, a relationship that in many ways sharpens the issue of the formalism of knowledge.

Talyzina describes the relationship of naïve and scientific concepts in controlled learning as follows: “In that regard, the prevailing naïve ideas were transformed before our eyes with the help of this technique; they rose to a new level. The child did not question the result obtained by the learned method if it contradicted his habitual conception. On the contrary, he stated and substantiated the fallacy of his previous conceptions (‘It turns out that the whale is not a fish; I thought it was a fish.’ If the experimenter then said: ‘But it lives in the sea like a fish,’ this did not confuse the subject: ‘It doesn’t matter that it lives in the sea. It feeds its young with milk, which means it is a mammal.’) Naïve concepts, having undergone a transformation, began to function with new content in the future” (Talyzina, 2018, pp. 230–231). But according to the described results of the study, it is not entirely clear in what sense the naïve concept is transformed and begins to function with new content and what role the statement of the fallacy of these naïve concepts plays here. This uncertainty was identified already in the early works of the author of the activity approach, A.N. Leontiev: “It is obvious that the generalization that lies behind the word ‘lever’ for the student, and the generalization that is the scientific concept of the lever, as it appears in the system of a given science, do not coincide.... In reality, this process [of the development of a generalization] obviously consists in the fact that the primary generalization behind the corresponding word, for example, behind the word ‘lever,’ develops in the child, is restructured, i.e., it rises to a new and higher level, and in the ideal case, it finally turns out to coincide with the generalization that is presented in the scientific concept of ‘lever’ ” (Leontiev, 2003, p. 325).

## The Problem of Transition from One System of Scientific or Naïve Concepts to Another

What happens to the student’s previously existing system during the mastery of a new system of concepts in the same subject area — whether the second one replaces the first or whether they begin to interact and if so in what form — this is the question that is most sharply posed when discussing the role of naïve concepts in the mastery of scientific ones.

A separate approach to “conceptual changes” arose as a reaction to the fact that many researchers and educators, when teaching scientific concepts to students, have encountered resistance from mundane thinking, “naïve concepts.” This approach identified such features of mundane thinking as resilience and systemicity (Keil, 1999; Vosniadou, 2019). Numerous studies using the “conceptual changes” approach have established how children’s thinking resists restructuring, remodeling, seems to reject externally introduced, alien singular concepts, and can be changed only as a whole, by “frameworks” or “theories”: “Conceptual development involves not just enrichment or elaboration of the existing knowledge systems but their considerable reorganization or restructuring. Conceptual change involves change in core concepts, conceptions, or conceptualizations (including rules, models, and theories). It concerns a large-scale restructuring of the existing knowledge system” (Inagaki & Hatano, 2013, pp. 195–196). At the same time, for this approach, the idea of the afunctionality of children’s thinking turned out to be dominant, that it does not have its own functions, but is only preliminary to adult thinking (cf. Romashchuk, 2008). This was reflected in the idea of “misconception,” and this expressed the attitude towards naive theories as having the exclusively negative role of offering “resistance” to the acquisition of scientific knowledge.

Although representatives of “conceptual changes” quite often refer to Vygotsky’s works, it is important to emphasize that he designated two variants for understanding the transition from one system of concepts to another. The first variant is the replacement of the old system (“structure”) of concepts with a new system. The second is the transformation of the old system into the new. The “late” Vygotsky advocated the transformation variant: “The child forms a new structure of generalization, first for a few concepts, usually newly acquired, for example, in the learning process; when he has mastered this new structure, by virtue of this alone he restructures and transforms the structure of all previous concepts” (Vygotsky, 1934, pp. 261–262). The new structure at the same time makes it possible “to move to a new and higher plane of logical operations. The old concepts, being involved in these operations of a higher type of thinking in comparison with the former ones, change by themselves in their structure” (Vygotsky, 1934). For example, a system of algebraic concepts is formed not alongside and not instead of a system of arithmetic ones, but through generalizations of the arithmetic concepts. For Vygotsky, the thesis that each scientific concept is included in a system (as structures of a special type) was especially important: a concept is like a cell in living tissue, not “like peas poured into a bag,” but is always included in a system of other concepts (Vygotsky, 1934).

That said, Vygotsky also finds reasons for the absence of these ideas in his study of the stages of development of syncretes and complexes (cf. Vygotsky, 1934, Chapter 4). The fact that in L.S. Sakharov’s study of artificial concepts, the stages of concept development were described as concentric circles rather than as a spiral of development (i.e., as complementing one another rather than as one stage superseding the previous one), according to Vygotsky himself, was influenced by the particular features of the methodology. A more complex form of transformation was demonstrated by J. Shif’s research (cf. Vygotsky, 1934, Chapter 6): the final stage in these experiments (the system of “true concepts”) was achieved not by displacing or replacing an naïve concept with a scientific one, but by simultaneously transforming both. The naïve concept is transformed into the form of the scientific concept, and the scientific is enriched with the content of the everyday.

Thus, the common element in the positions of Vygotsky and the “conceptual changes” approach is the understanding of children’s thinking as a complex, holistic formation that resists simple replacement by “adult” thinking. The main difference between these approaches lies in their analysis of the transition from naïve to scientific concepts. According to Vygotsky, the transition is carried out through a special type of transformation (“sublation”) of naïve concepts by scientific ones, and not by replacing the first with the second, as in the “conceptual changes” approach and the theories of the activity approach to learning. This completely changes the logic of the relationship between the two systems of concepts: the naïve and the scientific. In particular, Vygotsky rethought the phenomenon of the incorrectness of not only naïve, but even of scientific concepts, the “formalism” of which is a necessary means for transforming naïve concepts.

It is important to understand that behind Vygotsky’s theses here there is a more general methodological position, namely that of constructive criticism and an attitude towards the concept as the main form of this criticism.

## The Concept of ‘Constructive Criticism’: A Form of Criticism in L.S. Vygotsky’s Approach

Constructive criticism is closely associated with the dialectical category of “sublation” [aufheebung, Russian *sniatie* — translator's note], Vygotsky's analysis of which, seems to us, has not yet received its deserved attention in the large literature on Vygotsky (see, for example, synthesizing works such as Asmolov, 2022; Daniels et al, 2007; Veresov, 2020), and even those specialized works dedicated to Vygosky’s use of dialectics (e.g., Dafermos, 2015). First of all, Vygotsky uses the category of “sublation” fully consciously and deliberately: “It seems to us that in this case the relationship between the higher and lower forms may be best expressed by the recognition of what is usually called in dialectics sublation.... The double meaning of the German word “to sublate” must be recalled, says Hegel. By this word we mean first of all, ‘to eliminate,’ ‘to negate,’ and we say, according to this definition, that laws are repealed, ‘abolished,’ but the same word also means ‘to preserve,’ and we say that we will ‘preserve’ something. Using this word, we could say that elementary processes and the patterns governing them are buried in the highest form of behavior, i.e., they appear in it in a subordinate and hidden form” (Vygotsky, 1983a, pp. 113–114).

Vygotsky’s orientation toward the category of “sublation” begins already in his first major work, “The Psychology of Art” (Vygotsky, 1998), and found its expression in a special form of critique of psychological concepts produced in this work. In “The Psychology of Art,” Vygotsky’s method is analogous to that of K. Marx in “Capital” (see Il’enkov, 1997). Thus, in the first three theoretical chapters, Vygotsky criticizes three theories of the psychology of art: art as an influence of content, art as an influence of form, and art as an organization of catharsis in the psychoanalytic sense. Then, using examples of a fable, a short story, and a tragedy, Vygotsky deduces an idea of the cultural mechanism of art, which ensures the simultaneous experience of directly opposite emotions. This cultural mechanism is constructed according to the principle of the collision of “thesis” and “antithesis”: “From fable to tragedy, the law of aesthetic reaction is one: it contains affect that develops in two opposite directions, which at the final point, like in a short circuit, finds its annihilation” (Vygotsky, 1998, p. 275). For the emergence of two opposite emotions (and not, for example, one ambivalent one), he uses the opposition of the form and content of a work of art. Since content and form can both develop relatively independently of each other, this makes it possible to simultaneously bring directly opposite emotions to the highest degree of intensity, to a culmination, within which catharsis should occur as a liberation from the “violence” of each of the natural feelings through their “mutual destruction.” And then both the representatives of the objective school, concentrating on content, and the representatives of formalism, who emphasize the form, are partly right. But the representatives of psychoanalysis are also right, emphasizing the cathartic effect of art. All three of these criticized approaches are retained by Vygotsky in a sublated form. Vygotsky called this whole dynamic the three phases of aesthetic experience, the transformation of which moves a person from a passive to an active state. These three phases are easily correlated with the phases of “sublation”: “thesis” — “antithesis” — “synthesis.” Thus, according to Vygotsky’s logic, a cultured person achieves freedom, free emotional experience.

In his later works, Vygotsky ’deepened and made more complex a similar type of constructive criticism of previous theories and individual concepts. Suffice it to recall the main critical goal of “The Historical Meaning of the Crisis in Psychology” (Vygotsky, 1983): the main contradiction identified in this work between “mechanistic, physiological psychology” and “higher, spiritual psychology” found its resolution in the logic of sublation of “lower, natural mental functions” into “higher, cultural mental functions” described in “The History of the Development of Higher Mental Functions” (Vygotsky, 1983b). The importance of this type of criticism for Marxism was most succinctly pointed out by E.V. Il’enkov, emphasizing that for Marx, constructive criticism was the main method of thinking, constructing a theory, and analyzing economic facts: “So that the reconciliation of critical accounts with previously developed theories is not at all a side issue, not at all a matter of secondary importance, but a necessary form of development of the theory itself, the only possible form of theoretical analysis of real facts” (Il’enkov, 1997, p. 219). Constructive criticism is aimed at retaining the “rational core” of the previous theory and at the same time sifting out all its historically transient content (Il’enkov, 1997). Vygotsky as a Marxist pointed out the same constructive attitude toward criticism: “The matter does not end with the discovery of the barrenness of the principle, with criticizing it by pointing to curiosities and exaggerations at which schoolchildren point their fingers. In other words, the history of a principle does not end with its simple expulsion from a sphere that does not belong to it, with its simple rejection. After all, we recall that an alien principle penetrated science by a bridge of facts, real-life analogies; no one denied this. The time during which this principle grew stronger and more dominant increased the number of facts on which its imaginary power was based — partly false, partly true. The critique of these facts, the critique of the principle itself, brings new facts into the purview of science. It is not just a matter of facts: a critique must give its own explanation for the colliding facts; the theories assimilate each other and on this basis the regeneration of the principle takes place. Under the pressure of facts and alien theories, the newcomer changes its face” (Vygotsky, 1983a, pp. 355–356)

This type of criticism establishes a special form of transition from one theory to another, from one concept to another. But the question remains how relevant this form is not only for actual transitions in science, but also for the transitions between implicit and scientific theories in the learning process.

Of all the main theoreticians of the activity approach to learning, the clearest position was taken by V.V. Davydov: “Modern theoretical thinking in the process of its formation has assimilated the positive moments and means of empirical thinking, ‘sublated’ them in itself” (Davydov, 1972, p. 424). But in the curricula of developmental education, implementing the logic of sublation seems to have encountered certain problems. For example, an article by representatives of developmental education about the effectiveness of the transition from naïve to scientific concepts at the theoretical level, with reference to the positions of Vygotsky and Davydov, clearly formulates the thesis of the mutual enrichment of scientific and naïve concepts: “We are not talking about a scientific concept overcoming an naïve one, but about the intersection, the mutual enrichment of these two separate lines of development of conceptual thinking” (Davydov, 1972, p. 6). But enrichment apparently implies giving meaning to educational modeling by its inclusion in the form of a game: “Next, it will be shown that game heroes who embody the concepts being mastered first contribute to the emergence of a new quality of these concepts, and second become a support for the initiatives first-graders take with sound schemes” (Davydov, 1972, p. 7). Consequently, in the role of an naïve concept, the game form is used here, and not the content of any naïve concept. It is no coincidence that the article never mentions a single naïve concept, and does not even discuss their mutual enrichment with scientific concepts.

A.A. Margolis has presented a criticism of developmental learning similar to ours; he also sees the need for developmental education to have a fundamentally different understanding of “the relationship between the processes of learning and development, in which scientific concepts formed in the course of learning do not destroy and supplant the products of the development of the child’s own thought in the form of those spontaneous concepts with which he begins the learning process” (Margolis, 2020, p. 9). And that the neglect by proponents of developmental education of the integrity of the system of naïve concepts, which makes more justified the point of view of V.S. Bibler’s students that “in order to overcome, for example, a child’s naive idea of number and ways of dealing with number, it is necessary to construct this idea as something integral, as an opponent, to understand the basis of such an idea and to construct in the subject ways of overcoming just such an idea — and not simply to organize the learning of the ‘correct’ concept” (Margolis, 2020, p. 16). For all the detail and depth of the criticism provided in the article, it leaves unchanged the main point disputed by Vygotsky: it cannot be a question of overcoming children’s naive perceptions as such, but requires their sublation. It seems that an additional impetus to such efforts within the framework of developmental education may be provided by studying constructive criticism in the psychology of insightful problem solving.

## Constructive Criticism in Everyday Thinking: Thinking with Full Insight

Theoretical disputes and studies of insightful problem solving offer two variants for the mechanisms of this solution, one of which, “full insight,” is an analogue of the mechanism of constructive criticism described above.

Initially, through the opposition of the Gestalt psychology of thinking and A. Newell and G. Simon’s theory of problem space, insightful and regular problem solving were separated (cf., e.g., Metcalfe & Wiebe, 1987). Insightful problem solving was characterized by suddenness, a leap associated with the restructuring of a situation, and at the level of experience it was accompanied by an “aha-reaction,” whereas the processes for solving regular problems had exactly the opposite characteristics: a gradual sequence of steps toward a solution, without sudden breaks and strong emotions in the process. There is a trend in modern psychology of problem solving to combine these two types of problem solving. One of the most meaningful attempts at such a combination was made in representational change theory by S. Ohlsson and G. Knoblich (Knoblich et al., 1999; Ohlsson, 2011). The authors of this theory criticize the most established and traditional understanding of insight as a complete and correct solution of a problem that suddenly appears in the mind, and they distinguish two components in the insightful solution: overcoming the state of an impasse and the appearance in the mind of the final solution. Two mechanisms are most fundamental for this: constraint relaxation and chunk decomposition (Ohlsson, 2011). A necessary, but not sufficient condition for insight from the point of view of representational change theory is that the problem solver has reached an impasse: “Insights occur after the problem solver has encountered an impasse, i.e., a mental state in which problem solving has come to a halt; all possibilities seem to have been exhausted and the problem solver cannot think of any way to proceed” (Ohlsson, 1992, p. 4). An impasse occurs when, having tried all the possible solutions known to the solver, none of them could solve the problem; the impasse signals that there are no more ideas left about how the problem could be solved. The reason for the impasse is in the incorrect initial representation of the problem. The impasse prepares the conditions for the mechanisms of “constraint relaxation” and “chunk decomposition,” a restructuring of the faulty representation of the problem.

The authors of the theory, explaining the essence of the constraint relaxation mechanism, suggest that “problem solving might be less a matter of searching among possibilities than of redefining what to search for” (Knoblich et al., 1999, p. 1535). Using a simple example, they explain that if we need to break through a locked door, initially our actions are constrained by the idea that the door should remain intact, but then it may become necessary to look not for a key, but for an ax. In that case, the initial principle of the solution implied “opening the door,” and constraint relaxation allowed us to change the task to “breaking down the door.”

**Figure 1.**
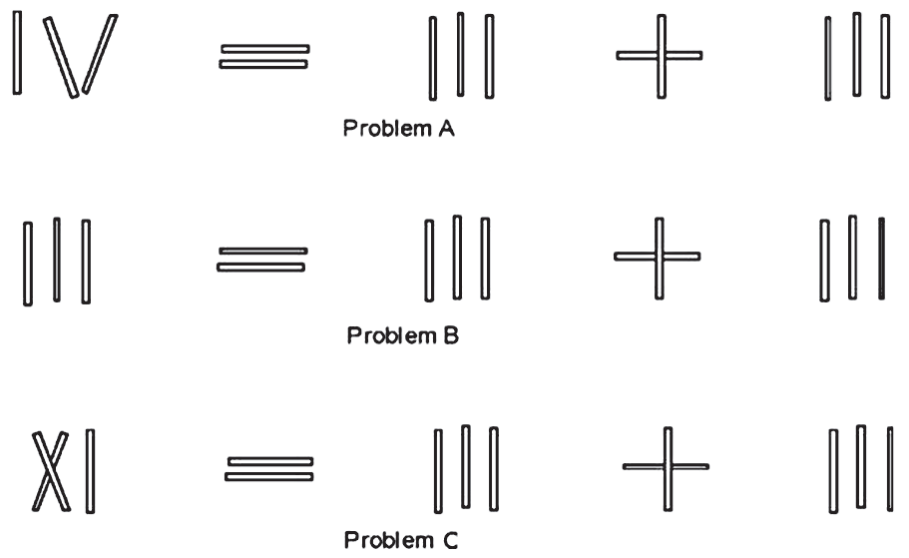
Arithmetic problems with matches

More specifically, the mechanisms of constraint relaxation and chunk decomposition can be explained by studying a series of matchstick numerical equality problems (Knoblich, 2001), which has become a classic within this approach. In all three tasks, the goal is formulated in the same way: to shift one match so that the equation becomes true (see *Figure 1*). The difference between the ease of solving problems is explained by the different density of chunks, which is provided by different strengths of the constraints.

The first problem, “A” (Fig. 1), is the easiest, since the decomposition of a chunk for the solution does not require the weakening of any constraint, but only the search for options (if you shift the match from “IV” to the right, you will have “VI”). The third problem, C, is more difficult, because a constraint appears, since it is necessary to decompose this chunk, whose parts do not have an independent value (you need to move one match from “XI” so that you get “VI”). But the most difficult task is “B,” since it requires weakening the constraint that determines the entire problem space associated with the requirement that only numbers need to be changed, but not operators (to solve it, you need to turn “=“ into “+”). Thus, “constraint” in representational change theory means the same thing as the concept of functional fixedness in Gestalt psychology, and insight is meant as a form of overcoming fixedness — from relaxing a small constraint to relaxing a constraint that shifts from one problem space to another, i.e., a fundamentally different representation of the problem.

But the similarity of representational change theory to the classical Gestalt psychology of thinking is largely illusory. First of all, for K. Duncker and M. Wertheimer, insight was not a sufficient criterion for thinking. Thus, Duncker wrote: “As a result of training, unexpected insight often arises. It then immediately becomes clear to the person what the required action should be, and the curve of errors drops sharply. Yet the principle of action itself may remain unknown. Paradoxical as it may seem, it turns out that a connection that is not fully accessible to insight may become insightful. This paradox is easily resolved if we keep in mind that here the concept of ‘insight’ is used with two different meanings. Grasping the common principle of a number of situations does not yet provide comprehension of this principle itself, its inner ‘why.’ What is comprehended is that once this common principle is given, specific situations must be just as they are and not otherwise” (Duncker, 1965, p. 177). Duncker distinguishes between two types of insight, one of which he later calls partial insight (or “insight of the second degree”), and the other full insight. Duncker associated thinking in the narrow sense only with full insight, and indicated that the main criterion for distinguishing between the two kinds of insight was an understanding of the inner “why” of the solution principle.

Thus, the generally accepted criteria for insight — suddenness, the “aha-reaction,” restructuring, and even finding the solution principle — are not, according to Duncker, criteria of thinking. To clarify, he gives a characteristic example: suppose the researcher gives the subject the task of guessing in which box the target object is hidden. The number and arrangement of boxes changes each time, but the researcher always uses the principle that the target object is hidden in the first box to the left of the middle of the entire group of boxes. As soon as the solver guesses the principle, he has an “epiphany” and will immediately solve the problems posed in the experiment. Since he understood the solution principle, but does not understand “why” this principle is a solution, this means that he has only a partial insight and an act of thinking has not occurred. But what is behind this “inner why,” how are we to understand the main criterion for distinguishing between the two types of insight?

For Duncker, the difference between thinking and deciding by means of trial and error lies in the concept of “understanding the directionality of the conflict”: “There is a fundamental difference between one *factor of conflict*, i.e., the presence of an action that does not lead to the desired result, and the *directionality of the conflict,* in which its nature is expressed” (Duncker, 1965, p. 37). The conflict is not just the absence of the desired result, but also an error in the method used to solve the problem (“action that does not lead to the desired result”). Thus, thinking begins with a critical analysis of an erroneous attempt to solve a problem. For clarification, he uses W. Köhler’s concept of “excessively” — when a chimpanzee tries to reach a banana with its paw, this conflict can lead to the beginning of an insightful solution if the chimpanzee stops looking for other options to get the banana and tries to understand why it failed to do so with its paws, and what is “too much” when using this method (its paws are too short). Such an analysis will begin to direct real thinking further, since the chimpanzee will be directed not so much to the fruit itself, as to “lengthening my arm.” It is with this analysis of the causes of the error that the concept of “why” is associated: “While the simple realization that ‘this doesn’t work’ can only lead ***to a direct variation of the old method***, the realization of ***why*** it does not work, the recognition of the ***basis of the conflict,*** results in a correspondingly definite ***variation,*** which corrects the recognized defect in the proposed solution” (Duncker, 1965, p. 107). Thus, for Duncker, the main stage of thinking as full insight is essentially connected with constructive criticism of one’s own erroneous way of solving a problem. Such criticism, which is aimed at understanding the reasons for the error, and its correction, leads to the transformation of the original method for solving the problem to a more suitable one (“a variation that corrects the perceived shortcoming of the proposed solution”).

A similar position in highlighting the role of constructive criticism of one’s own erroneous method of problem solving as the basis of the entire process of insightful problem solving was that of another major representative of the Gestalt psychology of thinking, M. Wertheimer (Wertheimer, 1987). His approach is especially valuable in that a significant part of his research involved problems used in school. Identifying the specifics of “thinking in the strict sense” as a holistically directed process, Wertheimer emphasizes the fundamental importance of understanding the disturbances that arise in solving a problem and striving to correct them: “Such a process is not a simple sum of individual steps, a set of operations that are not related to each other, but is a single process of thinking generated by the awareness of gaps in the situation, the desire to correct them, to fix what is bad, to achieve inner harmony” (Duncker, 1965, p. 79). Like Duncker, he indicates the connection between the disturbance and the erroneous action not too emphatically, but quite definitely.

**Figure 2.**
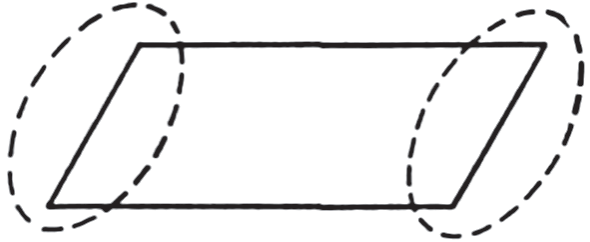
Locating the disturbance in finding the area of a parallelogram

So, for example, to clarify the concept of “disturbance,” Wertheimer taught students the solution of the problem of finding the area of a rectangle, and then gave a problem for the area of a parallelogram and registered whether an act of thinking occurs or not. Wertheimer gives the example of a girl who, in solving the given problem, demonstrated thinking. When she tries to use the area of a rectangle to solve the problem, she sees that the rectangle is only suitable for finding the area of the central part of the parallelogram, which means that this method does not directly lead to the correct solution. But then she begins to analyze the resulting disturbance and sees that using the area of the rectangle leads to an error because of the ends of the parallelogram (see *Figure 2*).

There are no right angles on the sides of the parallelogram, and the girl locates the disturbance in these two parts. And then the act of thinking is performed through an answer to the question of how to overcome the incorrect use of the area of a rectangle on a parallelogram. The girl’s thinking, directed by analysis of the disturbance, concludes that if both parts presenting the disturbance are combined — cutting off the triangular end of the parallelogram on one side and adding it to the other side — then you will have a rectangle again. And thus the girl derives the formula for the area of a parallelogram by transforming, “adapting” the formula for the area of a rectangle. This derivation of the method of solution fully answers the question of “why” the new formula is a solution to the problem of the area of a parallelogram: because it is associated with the transformation of a parallelogram into a rectangle, the formula for the area of which is known.

**Figure 3.**
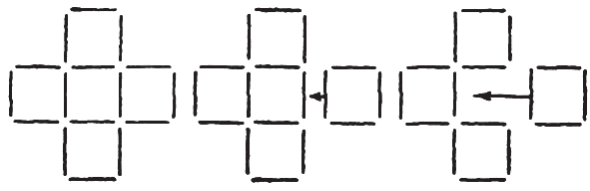
Katona’s “five squares” problem

The differences between the notion of “constraint” in representational change theory and the notion of “disturbance” in Gestalt psychology can be conveniently analyzed on the basis of Katona’s “Five Squares” problem, since both constraint and disturbance have been investigated with respect to it. Katona’s problem (see *Figure 3*) presents five squares arranged in the form of a cross, with the goal of rearranging three matchsticks to leave only four squares. The solution is to separate one square from the single structure. From the perspective of representational change theory, the onset of insight in this task is hindered by the constraint implicit in the subjects’ idea of constructing the four required squares in a single configuration (Öllinger et al., 2014).

If we solve this problem by understanding what the disturbance is, then the following option becomes possible (Emel’ianova, 2020). When trying to understand why you can’t manage to move three matches in the right way, you may encounter a contradiction: since each match is a side of a square, you need to rearrange the matches, reducing the squares, that is, *you have to reduce the number of squares without reducing the number of sides.* Understanding this disturbance leads us to examine the common, adjacent sides of the squares, which are the sources of the increase in the number of squares. In other words, with a holistically directed solution, we find that sides and matches are not the same thing, but there are common sides of squares, when one match forms the sides of two neighboring squares. The solution follows from the elimination of this disturbance: these common sides have to be separated. One of the principal consequences of using an understanding of the disturbance in solving the problem is that the resulting method of solution can be transferred to seemingly quite dissimilar problems (see Emel’ianova, 2020).

Thus, these two types of insight have exactly the opposite directionality. Partial insight emphasizes the interfering characteristics of erroneous trials, i.e., it identifies what the correct method of solution method should not possess, what “fixation” it should be free of. It is not by chance that the wording of the results of the analysis necessarily contain the negative particle “not” (the door should *not* be opened; in “match” numerical equations it is *not* necessary to change only the numerical values; you should *not* try to construct four triangles from six matches only on a plane, etc.). With full insight, on the other hand, something is revealed that an erroneous trial does not possess, but that a correct solution should have. This means that if we can see in constraint relaxation a critique of all the erroneous methods of a given problem space (the constraints on which must be weakened), then understanding the disturbance means precisely constructive criticism of a certain method of solving the problem, i.e., identifying what should be left in it and what should be changed to reach the right solution.

## Preconditions for Full Insight in the Developmental Education of D.B. El’konin-V.V. Davydov

There is a logic in developmental education that is similar to the logic of using the understanding of errors to produce full insight. It concerns the use of specially created obstacles to the development of understanding (a concept), which, in principle, is associated with learning activity as an activity to solve a learning problem: “The problem is unsolvable (‘I don’t know what to do, help me’) is reformulated into an underdetermined problem (‘I will be able to solve this problem, if…”). Here, where there is a contradiction between the method of action that the child already knows and the new problem conditions, the student formulates *knowledge of his own ignorance*, i.e., poses the actual learning task” (Davydov, 1996, p. 213). A feature of the technique for creating the obstacles necessary to create a learning task is that the obstacle, on the one hand, should lead to the impossibility of using the already mastered method of solving a problem, and, on the other hand, to a particular reaction to this impossibility. The student’s reaction should be not just rejection of the erroneous action and trying out other ones, but a particular thoughtful analysis of the situation. It is this analysis that transforms the problem from being, in Davydov’s words, unsolvable, to being underdetermined. In other words, analysis of the impossibility of using a certain method of solving a problem should help to form specific knowledge about the correct method (“I can solve this problem if ...”), and not just to provide the information that the method being used to solve the problem is wrong. Compared to the mechanisms of full insight, this technique of developmental education skips the stage of identifying the role of an erroneous solution and analyzing the causes of this erroneousness. Considering this technique from the standpoint of the Gestalt psychology of thinking helps to emphasize the concept of “error” behind such concepts as “obstacle,” “contradiction between the method of action and new conditions,” and, therefore, to provide for the possibility of using the logic of constructive criticism. Let’s look at this in a bit more detail.

This technique proposes the following sequential series of stages of transition from a practical to a learning task in the strict sense: 1) a practical task, 2) a “learning-practical task”, 3) a “learning-theoretical task”, 4) a learning task (Repkin & Repkina, 1997, pp. 199–208). A common methodology for shifting from one stage to the next is the creation of a special obstacle to solving the problem, which shifts the focus of the student from the final solution to overcoming this obstacle, and this overcoming becomes a problem of a new (the next) type. Thus, for example, such tasks as reading a word or finding the sum of numbers are practical problems. For the transition to the learning-practical stage, it is necessary, first of all, to provide preliminary training in a number of prerequisite actions, which are also practical in their result (e.g., separation of the sound shell of a word and its meaning, mastering the methods of analyzing the sound structure of a word and reflecting this structure in a graphic model). But secondly, and most importantly, it is necessary to introduce a complication to the conditions for implementation of these actions (for example, the presence of soft consonants in the word), which leads to the formation of a new intermediate goal associated with “the need to find out the connection between the conditions and methods of obtaining the result” (Davydov, 1996, p. 175).

Using such special obstacles in solving “learning-practical task”, the activities of the students include concepts and related methods of thoughtful analysis and generalization, which leads the student to the need to solve a “learning-theoretical task” (for example, to perform a phonemic analysis of the word). And through a new complication in the process of finding a solution, the student is faced with the need to identify ways to analyze the concept as a developing system, which characterizes the learning task and the full formation of learning activity. For example, when switching to morphemic analysis (a necessary prerequisite for phonemic analysis of a word and for solving a spelling task, since the solution to the problem “determining the phoneme in the strong position” depends on morphemic analysis), the students have to derive a system of particular concepts from the original concept of “parts of a word” (root, prefix, suffix, ending). “In other words, the methods of theoretical analysis of a concept serve as methods of constructing *systems* of concepts” (Repkin & Repkina, 1997, p. 206).

Thus, comprehension of the solution that arises when there are specially organized obstacles is the basis not just for deriving an individual concept, but for deriving through the construction of a system of concepts. The last thesis about the need for deriving the concept of building an integral system of concepts unites all three theories of the activity approach to learning that we are considering. But, first of all, none of them emphasizes the need for constructive criticism of the student’s mistakes, and, secondly, the system of concepts being built is not based on the transformation of another system of concepts through constructive criticism (up to implicit naive theories). The introduction of these two forms of constructive criticism can be substantiated both by Vygotsky’s theoretical analysis and by the experience of developing the psychology of insightful problem solving.

## Conclusion

The main thesis of the article is that mastery of a scientific concept is most effective when it is presented as a form of constructive criticism of another concept. This means that the concept should be mastered not simply by deriving it, transforming it from another concept (as they insist, for example, in developmental education, through derivation from a single concept, a concrete-universal, a “cell”), but by deriving it in the form of a special kind of criticism of the previous concept, i.e., in the form of sublation. On the other hand, criticism that necessarily arises in the learning process (for example, fixation on the student’s mistakes) is most productive when it occurs in a conceptual form, as constructive criticism of an implicit understanding, of a “naive” concept.

To substantiate this thesis, the problem of the formalism of scientific concepts in learning and the resistance of naïve concepts to attempts to replace them with scientific ones play an important role. Analysis of these two aspects of a single problem leads to the proposition that the introduction of scientific concepts requires an understanding of their systemic nature, i.e., their inclusion in a theory. And, therefore, it turns into a question of the interaction of theories — the one from which the new concept being studied is derived, and the one to which the opposing concept belongs. Naïve concepts accentuate this by the fact that they represent one “naive” theory or another, and, moreover, it was precisely when discussing the relationship between scientific and naïve concepts that Vygotsky put forward the thesis about the need to transform the system of concepts, and not just to replace one with another. The philosophy of science, primarily based on German classical philosophy, suggests a solution in the concept of “constructive criticism.” The psychology of thinking, through the contrast of partial and full insight with the simpler material of insightful problem solving that is much more widespread in everyday life, in turn reinforces the idea of constructive criticism as the main method for deriving a new way of solving a problem, i.e., a potential concept. The constructiveness of criticism in this case consists in understanding the reason for an error of inappropriate action in order to correct the shortcoming of the method and transform it into an appropriate one. A holistic combination of all three approaches — the activity approach to learning, Vygotsky’s approach, and the approach of the psychology of insightful problem solving — makes it possible theoretically and methodologically to concretize a position on mastery of a scientific concept as a form of constructive criticism.

## References

[ref1] Asmolov, A.G. (2022). The Historical Meaning of the Crisis of Cultural Activity Psychology. Journal of Russian & East European Psychology, 59(1–3). 10.1080/10610405.2022.2115783

[ref2] Dafermos, M. (2015). Otrazhenie otnoshenii mezhdu kulturno-istoricheskoi teoriei i dialektikoi [Reflection on the relationship between cultural-historical theory and dialectics]. Psikhologicheskaia nauka i obrazovanie [Psychological science and education], 20(3), 16–24. 10.17759/pse.2015200303

[ref3] Daniels, H., Cole, M., & Wertsch, J. V. (Eds.) (2007). The Cambridge companion to Vygotsky. Cambridge University Press. 10.1017/CCOL0521831040

[ref4] Davydov, V.V. (1972). Vidy obobshchenii v obuchenii (logiko-psikhologicheskie problemy postroeniia uchebnykh predmetov) [Types of generalizations in instruction (logical and psychological problems of constructing academic subjects)]. Pedagogy.

[ref5] Davydov, V.V. (1996). Teoriia razvivaiushchego obucheniia [Developmental learning theory]. INTOR.

[ref6] Duncker, K. (1965). Kachestvennoe (eksperimentalnoe i teoreticheskoe) issledovanie produktivnogo myshleniia [Qualitative (experimental and theoretical) research on productive thinking]. In A.M. Matyushkina (Ed.), Psikhologiya myshleniia [The psychology of thinking]. Progress.

[ref7] Emel’ianova, A.S. (2020). Rol’ ponimaniia oshibki resheniia v vozniknovenii ‘polnogo insayta’ [The role of understanding problem-solving errors and the emergence of ‘full insight’]. [Specialist Diploma Thesis, Lomonosov Moscow State University].

[ref8] Galperin, P.Ya. (2002). Lektsii po psikhologii: uchebnoe posobie [Lectures on psychology: textbook]. Higher School.

[ref9] Il’enkov, E.V. (1997). Dialektika abstraktnogo i konkretnogo v nauchno-teoreticheskom myshlenii [Dialectic of the abstract and the concrete in scientific theoretical thinking]. ROSSPEN

[ref10] Inagaki, K., & Hatano, G. (2013). Conceptual change in naive biology. In S. Vosniadou (Ed.), International handbook of research on conceptual change (pp. 195–219). Taylor and Francis.

[ref11] Keil, F..C. (1999). Conceptual change. In R.A. Wilson & F.C. Keil (Eds.), The MIT encyclopedia of the cognitive sciences (pp. 179–182). MIT Press.

[ref12] Knoblich, G., Ohlsson, S., Haider, Y., & Rhenius, D. (1999). Constraint relaxation and chunk decomposition in insight problem solving. Journal of Experimental Psychology: Learning, Memory, and Cognition, 1534–1555. https://www.hf.uni-koeln.de/data/fgpsych/File/Haider/Knoblich_etal_1999.pdf

[ref13] Knoblich, G.O., Ohlsson, S., & Raney, G.E. (2001). An eye movement study of insight problem solving. Memory and Cognition, Oct. 29(7), 1000-9. 10.3758/BF03195762 11820744

[ref14] Leontiev, A.N. (2003). Stanovlenie psikhologii deiatel’nosti: Rannie raboty [The formation of activity psychology]. Smysl.

[ref15] Margolis, A.A. (2020). Zona blizhayshego razvitiia (ZBR) i organizatsiia uchebnoi deiatel’nosti uchashchikhsia [The zone of proximal development (ZPD) and the organization of students’ learning activity]. Psikhologicheskaia nauka i obrazovanie [Psychological science and education], 25(4), 6–27. 10.17759/pse.2020250402

[ref16] Metcalfe, J., & Wiebe, D. (1987). Intuition in insight and noninsight problem solving. Memory & Cognition, 15(3), 238–246. 10.3758/BF03197722 3600264

[ref17] Ohlsson, S. (1992). Information-processing explanations of insight and related phenomena. In M. Keane & K. Gilhooly (Eds.), Advances in the psychology of thinking (pp. 1–44). Harvester-Wheatsheaf.

[ref18] Ohlsson, S. (2011). Deep learning: How the mind overrides experience. Cambridge University Press.

[ref19] Öllinger, M., Jones, G., &Knoblich, G. (2014). Insight and search in Katona’s five-square problem. Experimental Psychology, 61(4), 263–272. 10.1027/1618-3169/a000245 24351983

[ref20] Repkin, V.V. & Repkina, N.V. (1997). Razvivaiushchee obuchenie: teoriia i praktika [Developmental education: theory and practice]. Peleng.

[ref21] Romashchuk, A.N. (2008). Evoliutsionizm v ponimanii razvitiia i ego preodolenie L.S. Vygotskim [Evolutionism in the undersanding of development and its overcoming by L.S. Vygotsky]. Kulturnoistoricheskaia psikhologiia [Cultural-historical psychology], 2, 50–59. https://psyjournals.ru/journals/chp/archive/2008_n2/chp_2008_n2_Romashchuk.pdf?ysclid=llcabifuna143197274

[ref22] Talyzina, N.F (1995). Formirovanie matematicheskikh ponyatii [The formation of mathematical concepts]. In N.F. Talyzina (Ed.), Formirovaniye priemov matematicheskogo myshleniia [The formation of techniques in mathematical thinking]. Ventana-Graf.

[ref23] Talyzina, N.F (2018). Deiatel’nostnaia teoriia ucheniia [The activity-based theory of learning]. Moscow University Press.

[ref24] Tsukerman, G.A., Obukhova, O.L., Ryabinina, L.A., & Shibanova, N.A. (2017). Vvedenie iskhodnykh poniatii: v poiskakh nedostaiushchikh opor [Introducing basic concepts: In search of the missing scaffolds]. Kulturno-istoricheskaia psikhologiia [Cultural-historical psychology], 13(4), 4–14. 10.17759/chp.2017130401

[ref25] Veresov, N.N. (2020). Chto nam delat’ s ‘Bolshoi Korolevskoi Pechatiu’: novaia realnost’ v nasledii Vygotskogo [Discovering the ‘Great Royal Seal’: new reality of Vygotsky’s legacy]. Kulturno-is-toricheskaia psikhologiia, 16(2), 107–117. 10.17759/chp.2020160212

[ref26] Vosniadou, S. (2019). The development of students’ understanding of science. Frontiers in Education, 4. 10.3389/feduc.2019.00032

[ref27] Vygotsky, L.S. (1998). Psikhologiia iskusstva [The psychology of art]. Feniks.

[ref28] Vygotsky, L.S. (1983a). Istoricheskii smysl psikhologicheskogo krizisa [The historical meaning of the crisis in psychology]. In L.S.Vygotskii Sobr. soch. v shesti tomakh. T. 1 [L.S. Vygotsky. Collected works in six volumes, Vol. 1] (pp. 292–436). Pedagogika.

[ref29] Vygotsky, L.S. (1983b). Istoriia razvitiia vysshikh psikhicheskikh funktsii. In L.S. Vygotskii Sobr. soch. v shesti tomakh. T. 3 [L.S. Vygotsky. Collected works in six volumes, Vol. 3] (pp. 5–328). Pedagogika.

[ref30] Vygotsky, L.S. (1934). Myshlenie i rech’ [Thinking and language]. Poligrafkniga.

[ref31] Wertheimer, M. (1987). Produktivnoe myshleniye [Productive thinking]. Progress.

